# Impact of the Inoculation Method of *Geotrichum candidum*, Used as Biocontrol Agent, on T-2 Toxin Produced by *Fusarium sporotrichioides* and *F. langsethiae* during the Malting Process

**DOI:** 10.3390/toxins14040239

**Published:** 2022-03-26

**Authors:** Hiba Kawtharani, Sandra Beaufort, Philippe Anson, Patricia Taillandier, Florence Mathieu, Selma Pascale Snini

**Affiliations:** Laboratoire de Génie Chimique, Université de Toulouse, CNRS, INPT, UPS, 31326 Toulouse, France; hiba.kawtharani@toulouse-inp.fr (H.K.); sandra.beaufort@toulouse-inp.fr (S.B.); philippe.anson@toulouse-inp.fr (P.A.); patricia.taillandier@toulouse-inp.fr (P.T.)

**Keywords:** T-2 toxin, *G. candidum*, phenyllactic acid, *F. sporotrichioides*, *F. langsethiae*, biocontrol agent, mycotoxin, barley, micro-malting

## Abstract

In malt production, steeping and germination steps offer favorable environmental conditions for fungal proliferation when barley is already contaminated by *Fusarium* species, T-2 toxin producers. However, the use of *G. candidum* as a biocontrol agent can prevent this proliferation. Indeed, in previous work, a correlation between phenyllactic acid (PLA) production by *G. candidum* and the reduction in *Fusarium sporotrichioides* and *F. langsethiae* growth and T-2 toxin concentration was demonstrated. In the present study, to improve the efficiency of *G. candidum*, the effects of the inoculum concentration and the inoculation method of *G. candidum* on PLA and T-2 toxin concentrations were evaluated. First, co-culture experiments with *Fusarium* species and *G. candidum* were conducted in a liquid synthetic medium. The results showed that inoculation of *G. candidum* in the freeze-dried form at 0.4 g/L allowed the production of PLA from the second day of incubation associated with a reduction in T-2 toxin concentration of 82% and 69% produced by *F. sporotrichioides* and *F. langsethiae,* respectively. Moreover, the activated form of *G. candidum* at 0.4 g/L enhanced PLA concentration leading to better T-2 toxin reduction. Second, experiments were conducted on artificially infected barley kernels with both *Fusarium* species under conditions mimicking the malting step. As for co-culture experiments, the use of the activated form of *G. candidum* was established as the best condition for T-2 toxin concentration reduction for a 3 day malting period.

## 1. Introduction

Cereal crops are highly susceptible to fungal contamination, and barley crops are no exception. Barley is used to produce malt, which is the main ingredient in beer production along with water, hops and yeast. The quality of barley kernels is decisive for the sanitary quality and the marketing of the beer, which is the most consumed alcoholic beverage in the world. Thus, barley contamination can be a source of public health concern, especially for heavy consumers [1,2,3]. Fungal infection of barley kernels occurs in the field and is often associated with *Fusarium* species, which are phytopathogenic fungi. Indeed, this genus is held responsible for the *Fusarium Head Blight* disease [4], resulting in quality deterioration of grain. Moreover, most of these fungi can produce toxic secondary metabolites, called mycotoxins. According to Magan et al., applying fungicides to crops can limit *Fusarium* spp. proliferation. However, mycotoxin contamination can remain intact [5]. 

In malt production, the first two stages (steeping and germination) offer favorable conditions for fungal proliferation from barley already contaminated by fungal spores. Indeed, nutrients abundance, grain moisture, temperature and process duration can enhance both fungal growth and mycotoxin production [6,7]. Then, during the kilning, temperature increases progressively but rarely exceeds 85 °C. Thus, as mycotoxins are thermostable, they persist even at the end of the production process [8]. In 2013, Inoue et al. evaluated the fate of mycotoxins during beer brewing and fermentation from spiked malt by several mycotoxins, including trichothecenes. They demonstrated that deoxynivalenol (DON), nivalenol (NIV) and T-2/HT-2 toxins (T-2/HT-2) should be carefully controlled due to their toxicity and residual content being higher than other mycotoxins [9]. Other studies analyzed beer products in Europe and came to the same conclusion: new European regulations and recommendations that limit the maximum level of these common mycotoxins in marketed beer products are needed to guarantee consumer safety [10,11,12]. Among these mycotoxins, T-2 toxin, type-A trichothecenes, is produced in warm and moist conditions mainly by *F. langsethiae, F. poae* and *F. sporotrichioides* [4,13]. It is the most cytotoxic of type A trichothecenes and has adverse effects on cellular metabolism [14,15]. Despite the many effects observed in animals, there is only limited evidence for the carcinogenicity of T-2 toxin [15]. Therefore, the IARC classified the molecule in group 3 as not classifiable as to its carcinogenicity to humans [16]. In cereals and cereal-based products, the European Union (EU) proposed recommendations with a maximum limit in barley (including malting barley) of 200 µg/kg [17].

Several biological, chemical and physical strategies applied before and/or after harvest are proposed to reduce the waste of food and agricultural commodities associated with fungal spoilage and/or mycotoxins contamination. Among these strategies, biocontrol methods may be of interest, especially those that involve the use of microorganisms to reduce these contaminations. In this context, the French Institute of Brewing and Malting (IFBM) filed a patent that proposes the use of a *Geotrichum candidum* strain, a filamentous yeast, during the malting step. *G. candidum* is frequently used in the agro-food industry as it takes part in certain foodborne microflora. Indeed, commercial *G. candidum* strains are employed as a starter culture for cheese ripening or in the brewing process to give the beer a distinctive floral taste. Moreover, *G. candidum* has not been listed as a human pathogen but may be involved in the aqueous acid rot of citrus. The *G. candidum* strain used in the present study (IFBM Malting Yeast^®^) was specifically selected for its ability to reduce the development of undesirable flora such as *Fusarium* spp. and to reduce the contamination of beer produced by the T-2 toxin [18]. However, until recently, little work has been published on the interactions between *G. candidum* and *Fusarium* species. First, Gastellum et al. (2012) demonstrated that *G. candidum* partially inhibited *F. langsethiae* growth leading to the reduction in T-2 toxin concentration [19]. Then, Kawtharani et al. (2020) complemented these results and demonstrated that *G. candidum,* inoculated at 0.2 g/L under the activated form, produced phenyllactic acid (PLA) in the early stages of growth and that PLA was responsible for the reduction in *Fusarium* growth as well as the reduction in T-2 toxin concentration. Indeed, T-2 toxin concentration and fungal growth were reduced by almost 90% when PLA reached its highest value after 24 h of incubation [20]. 

Currently, maltsters mix the *G. candidum* strain under freeze-dried form during the steeping, the first step of the malting process, conducted between 10 and 25 °C during 30–50 h. Then, during the germination step (for three to five days), *G. candidum* colonizes barley kernels to avoid fungal proliferation.

The objective of the present study was to evaluate the impact of the inoculation method (i.e., concentration and activated form or freeze-dried form) of *G. candidum* strain on two *Fusarium* species in terms of fungal growth and T-2 toxin concentration. Thus, two types of experiments were conducted using *G. candidum* under freeze-dried or activated forms at two concentrations (0.2 and 0.4 g/L). First, experiments were conducted in vitro in a liquid synthetic medium, and second, in conditions mimicking the malting step. In both experiments, PLA and T-2 toxin concentrations were monitored to evaluate the efficiency of *G. candidum*.

## 2. Results

### 2.1. G. candidum Growth and PLA Production Kinetics

**Figure 1:**
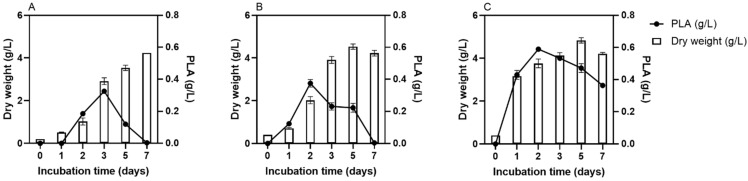
*G. candidum* dry weight (g/L) and PLA concentration (g/L) in pure culture. *G. candidum* was inoculated under freeze-dried form at 0.2 g/L (**A**), under freeze-dried form at 0.4 g/L (**B**) and under activated form at 0.4 g/L (**C**).

Figure 1 presents the *G. candidum* dry weight and the PLA concentration obtained under the different pure culture conditions. When *G. candidum* was inoculated under freeze-dried form at 0.2 g/L and 0.4 g/L, the dry weight gradually increased to reach a maximum value of 3.5 g/L (±0.1) and 4.5 g/L (±0.1) at five days, respectively (Figure 1A,B). However, when *G. candidum* was inoculated under activated form at 0.4 g/L, the dry weight increased abruptly within 24 h to reach a value of 3.2 g/L (±0.01) and then increased slowly to attain a maximum value of 4.8 g/L (±0.1) after five days of incubation (Figure 1C). Regarding PLA concentrations, when *G. candidum* was inoculated under freeze-dried form at 0.2 g/L, the highest PLA concentration was reached at the third day of incubation time (0.33 g/L ± 0.01), whereas when it was inoculated under freeze-dried form at 0.4 g/L, the highest PLA concentration was reached at the second day of the experiment (0.37 g/L ± 0.02). Then, PLA concentrations decreased until reaching the null value after 7 days of incubation (Figure 1A,B). However, when *G. candidum* was inoculated under activated form at 0.4 g/L, the highest PLA concentration was reached on the second day of the experiment (0.59 g/L ± 0.01) and remained high until the end of the experiment (0.36 g/L ± 0.01) (Figure 1C).

Since *G. candidum* growth values were slightly different from one condition to another, the PLA-specific productions were calculated and presented in Table 1. Results demonstrated that the PLA was highly accumulated in the medium between the first and second days in all culture conditions. Whatever the inoculum concentration, when *G. candidum* was inoculated under the freeze-dried form, PLA specific production was drastically reduced after three days of incubation. However, when *G. candidum* was inoculated under the activated form, PLA-specific production remained high even after three days of incubation.

**Figure 2:**
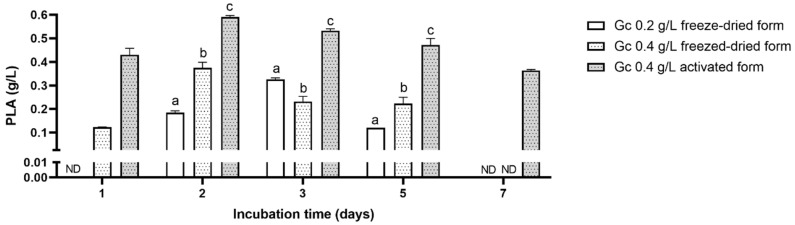
Comparison of PLA concentration (g/L) in *G. candidum* pure cultures. *G. candidum* was inoculated in Ym medium under freeze-dried or activated form at 0.2 or 0.4 g/L and incubated for up to 7 days. PLA concentrations (g/L) were measured at several incubation times (Two-way ANOVA; Tukey’s multiple comparisons post hoc test; comparisons were made for each incubation time between the different inoculation conditions; data with different letters are significantly different *p*-value < 0.05). ND = not detected.

Figure 2 shows the comparison of PLA concentrations as a function of the *G. candidum* inoculation method. When *G. candidum* was inoculated under the freeze-dried form at 0.2 g/L, the highest concentration of PLA was obtained on the third day of incubation and reached 0.325 g/L (±0.007). However, when it was inoculated at 0.4 g/L, the highest PLA concentration was obtained on the second day of incubation and reached 0.37 g/L (±0.02). In addition to that, PLA disappeared from the medium after five days of incubation time in both conditions. However, inoculating *G. candidum* under activated form at 0.4 g/L led to obtaining a maximal value of PLA at two days and helped to maintain PLA values high during the whole incubation time. Moreover, the maximum value reached at two days was higher than when *G. candidum* was inoculated under the freeze-dried form.

### 2.2. Co-Culture between G. candidum and Fusarium Strains in Ym Synthetic Medium 

**Figure 3:**
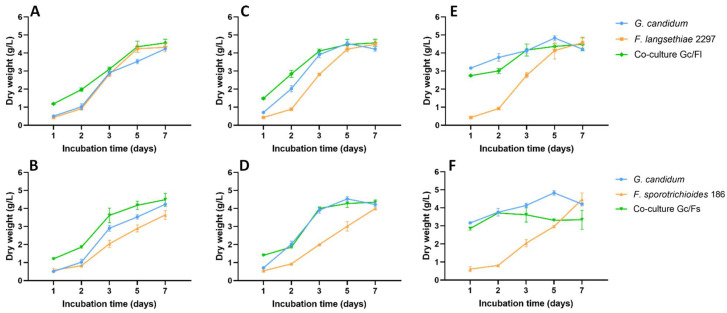
Microbial dry weights (g/L) in pure cultures and in co-culture experiments of *G. candidum* with *F. langsethiae* 2297 (*Gc/Fl*) or *F. sporotrichioides* 186 (*Gc/Fs*). *G. candidum* was inoculated under freeze-dried form at 0.2 g/L (**A**,**B**), under freeze-dried form at 0.4 g/L (**C**,**D**) and under activated form at 0.4 g/L (**E**,**F**).

Co-culture experiments were conducted by using the *G. candidum* strain either under the freeze-dried form or under activated form inoculated at 0.2 or 0.4 g/L with *F. langsethiae* 2297 (*Gc/Fl*) or with *F. sporotrichioides* 186 (*Gc/Fs*). In such conditions, the weight of both microorganisms could not be distinguished. Thus the total microbial dry weight was measured in g/L, and results are presented in Figure 3. In *Fusarium* pure cultures, fungal biomass increased throughout the experimentation. Indeed, after one day of incubation time, the average dry weight of *F. langsethiae* 2297 and *F. sporotrichioides* 186 were 0.43 g/L (±0.30) and 0.58 g/L (±0.08) and reached 4.46 (±0.24) and 4.03 (±0.42), respectively until the end of the experiment. In all co-culture conditions, the total microbial dry weights were always less than the sum of dry weights obtained separately in pure cultures. Indeed, when *G. candidum* was inoculated under freeze-dried form at 0.2 g/L, the total microbial dry weights were 4.57 g/L (±0.21) in *Gc/Fl* (Figure 3A) and 4.49 g/L (±0.36) in *Gc/Fs* (Figure 3B). When *G. candidum* was inoculated under freeze-dried form at 0.4 g/L, the total microbial dry weights were 4.57 g/L (±0.21) in *Gc/Fl* (Figure 3C) and 4.35 g/L (±0.13) in *Gc/Fs* (Figure 3D). Finally, when *G. candidum* was inoculated under activated form at 0.4 g/L, the total microbial dry weights were 4.49 g/L (±0.36) in *Gc/Fl* (Figure 3E) and 3.34 g/L (±0.52) in *Gc/Fs* (Figure 3F).

**Figure 4:**
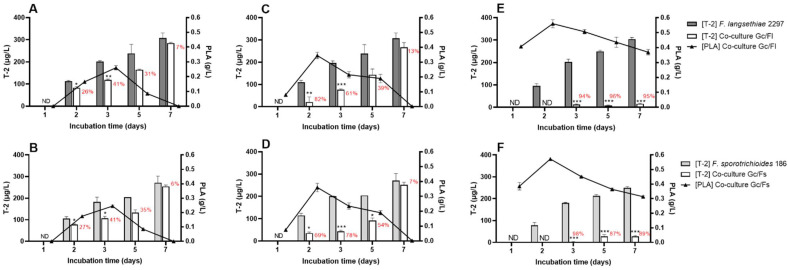
T-2 toxin concentration (µg/L) and PLA concentration (g/L) in co-culture experiments of *G. candidum* with *F. langsethiae* 2297 (*Gc/Fl*) or *F. sporotrichioides* 186 (*Gc/Fs*). *G. candidum* was inoculated under freeze-dried form at 0.2 g/L (**A**,**B**), under freeze-dried form at 0.4 g/L (**C**,**D**) and under activated form at 0.4 g/L (**E**,**F**). T-2 toxin reduction percentages were calculated and noted in red. One-way ANOVA, Tukey’s multiple comparisons post hoc test, * *p*-value < 0.05; ** *p*-value < 0.01 *** *p*-value < 0.01. ND = not detected.

Figure 4 shows T-2 toxin and PLA concentrations for each co-culture experiment.

Concerning T-2 toxin concentration, when *G. candidum* was inoculated under the freeze-dried form at both concentrations, in co-culture with *F. langsethiae* 2297 (*Gc/Fl*) or with *F. sporotrichioides* 186 (*Gc/Fs*), T-2 toxin was detected on the second day of incubation at lower concentrations than in both *Fusarium* pure cultures. Furthermore, T-2 toxin concentration increased up to seven days to a value insignificantly different from that observed in *Fusarium* pure cultures (Figure 4A–D). When *G. candidum* was inoculated under activated form at 0.4 g/L, in co-culture with *F. langsethiae* 2297 (*Gc/Fl*) or with *F. sporotrichioides* 186 (*Gc/Fs*), T-2 toxin was detected after three days of incubation at very low concentrations (11.8 µg/L ± 2.6 in *Gc/Fl* and 2.7 µg/L ± 1.1 in *Gc/Fs*). Moreover, values remained low after seven days of incubation time (15.8 µg/L ± 1.2 in *Gc/Fl* and 28.4 µg/L ± 2.2 in *Gc/Fs*) in comparison with T-2 toxin concentration in *F. langsethiae* 2297 (304.20 µg/L ± 7.92) and *F. sporotrichioides* 186 (249.98 µg/L ± 5.77) pure cultures (Figure 4E,F). In order to ensure that T-2 toxin was not converted, HT-2 toxin was also monitored and was not detected. Based on T-2 toxin concentrations measured in co-culture experiments, T-2 toxin reduction percentages were calculated for each culture condition and are also presented in Figure 4. When *G. candidum* was inoculated under freeze-dried form at 0.2 or 0.4 g/L, the T-2 toxin reduction percentage decreased from the third day of incubation. However, when *G. candidum* was inoculated under activated form at 0.4 g/L, the T-2 toxin reduction percentage remained high throughout the experiment (95% in *Gc/Fl* and 89% in *Gc/Fs* after seven days of incubation). 

Regarding PLA concentrations in co-culture experiments where *G. candidum* was inoculated under freeze-dried form at 0.2 g/L, the PLA concentration highly increased during the first three days to reach the highest value of 0.26 g/L (±0.01) in *Gc/Fl* and 0.24 g/L (±0.01) in *Gc/Fs* (Figure 4A,B). When the concentration of *G. candidum* was increased to 0.4 g/L, the highest PLA concentration was attained earlier, on the second day of incubation to reach 0.35 g/L (±0.01) in *Gc/Fl* and 0.36 g/L (±0.01) in *Gc/Fs* (Figure 4C,D). Afterward, in both co-culture conditions, PLA concentration decreased until reaching the null value after seven days of incubation. Otherwise, when *G. candidum* was inoculated under activated form at 0.4 g/L, in both co-culture experiments, PLA concentrations were maximal with 0.56 g/L (±0.02) in *Gc/Fl* and 0.57 g/L (±0.03) in *Gc/Fs* at two days of incubation and remained high until the end of the experiment (Figure 4E,F).

### 2.3. Micro-Malting Assays 

Micro-malting assays were conducted with *F. langsethiae* 2297 or with *F. sporotrichioides* 186 to mimic the germination step during the malting process and were held three and five days, as was performed by maltsters in beer industries. 

**Figure 5:**
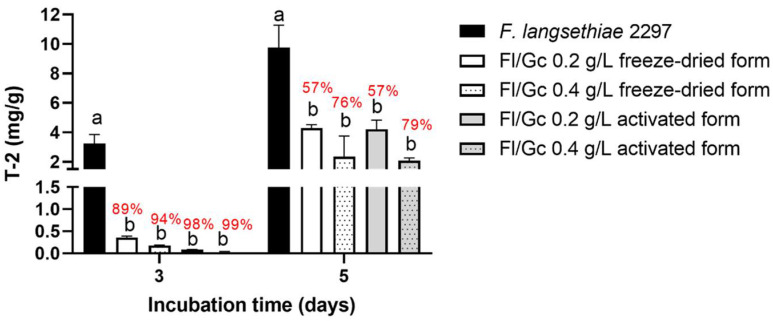
T-2 toxin concentration (mg/L) in micro-malting experiment with *F. langsethiae* 2297. *G. candidum* was inoculated at 0.2 or 0.4 g/L under freeze-dried or activated form (Two-way ANOVA, Tukey’s multiple comparisons post hoc test, comparisons were made for each incubation time between the different inoculation conditions data with different letters are significantly different *p*-value < 0.05). T-2 toxin reduction percentages were calculated and noted in red.

In the case of *F. langsethiae* 2297, T-2 toxin concentration and T-2 toxin reduction percentage for each experimental condition are presented in Figure 5. The inoculation of *G. candidum* under the freeze-dried form or activated form either inoculated at 0.2 or 0.4 g/L significantly reduced the final T-2 toxin concentration at the two incubation times. Depending on the experimental condition and the incubation time, the percentage of toxin reduction was between 57% and 99%. The best reduction values were obtained at three days of incubation (between 89% and 99%) whatever the *G. candidum* inoculated form. Although the reduction decreased at five days of incubation, it was still between 76% and 79% when inoculated with *G. candidum* at 0.4 g/L under freeze-dried or activated form.

**Figure 6:**
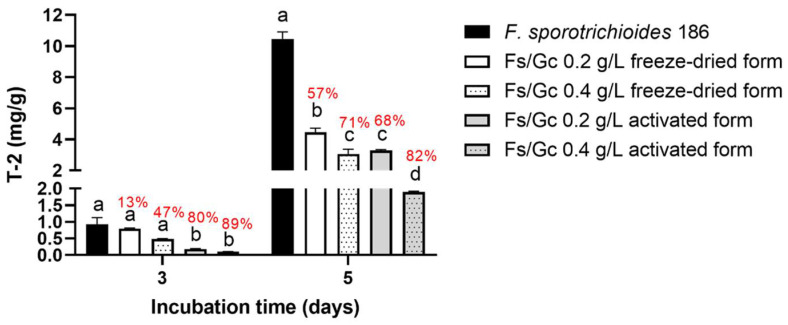
T-2 toxin concentration (mg/L) in micro-malting experiment with *F. sporotrichioides* 186. *G. candidum* was inoculated at 0.2 or 0.4 g/L under freeze-dried or activated form (Two-way ANOVA, Tukey’s multiple comparisons post hoc test, comparisons were made for each incubation time between the different inoculation conditions, data with different letters are significantly different *p*-value < 0.05). T-2 toxin reduction percentages were calculated and noted in red.

For the micro-malting experiment with *F. sporotrichioides* 186, T-2 toxin concentration and T-2 toxin reduction percentage for each experimental condition are presented in Figure 6. At three days of incubation, only the activated forms of *G. candidum* can significantly reduce the final T-2 toxin concentration in comparison with the control condition (T-2 toxin reduction percentage between 80 and 89%). At five days of incubation, T-2 toxin concentrations decreased significantly in all experimental conditions, and the T-2 toxin reduction percentage ranged between 57 and 82% depending on the *G. candidum* inoculation method. However, the most efficient results were obtained when *G. candidum* was inoculated at 0.4 g/L, which led to a T-2 toxin reduction percentage of 82%. 

PLA concentrations were also monitored and are detailed in Table 2. In micro-malting experiments where *G. candidum* was inoculated under activated form at three days of incubation, PLA production was better than when *G. candidum* was inoculated under the freeze-dried form. Moreover, the inoculation of *G. candidum* at 0.4 g/L under activated form was the only condition that allowed a high concentration of PLA at five days of incubation.

## 3. Discussion

During the brewing process, it is known that the malting step provides the best conditions (22 °C and high humidity) for *Fusarium* development and T-2 toxin production [6,7]. In order to reduce the development of undesirable microflora and, more particularly, *Fusarium* species, the French Institute of Brewing and Malting recommends the addition of the *G. candidum* X16010211 strain during the malting step [18]. Thus, such conditions significantly reduced the presence of filamentous fungi belonging to *Fusarium, Aspergillus* and *Rhizopus* genera [21]. Better yet, its use lowered worth viscosity and thus improved the physical, chemical and organoleptic characteristics of the beer. Using starter cultures as a means to limit the development of undesirable microflora and to eliminate the potential presence of unfavorable fungi metabolite is considered as a replacement of chemical agents used for grain disinfection during the brewing process [22]. 

In a previous study, the interaction mechanisms between two *Fusarium* species and *G. candidum* were deciphered in Ym synthetic medium at 22 °C [20]. The reduction in fungal biomass and the T-2 toxin concentration were both correlated to the production of PLA by *G. candidum.* Indeed, *G. candidum* was inoculated under activated form at 0.2 g/L, and the highest PLA concentration was obtained on the third day of incubation and reached 0.41 g/L (±0.03). However, nowadays, brewers inoculate *G. candidum* under the freeze-dried form with humid barley kernels and leave it for three to five days. Thus, the present study aimed to evaluate the influence of the *G. candidum* inoculum method in terms of form (i.e., freeze-dried or activated form) and concentration in the in vitro experiments in Ym synthetic medium and at the micro-malting scale. 

Experimentations conducted in Ym synthetic medium showed that PLA production was highly dependent on the inoculation method of *G. candidum.* Indeed, when *G. candidum* was added in the freeze-dried form at 0.2 g/L, PLA production was delayed and was detected after 48 h towards 24 h when *G. candidum* was added in the freeze-dried or in activated forms at 0.4 g/L. Moreover, whatever the *G. candidum* concentration, the PLA concentration reached lower values when it was inoculated in freeze-dried form. However, the final dry weight of *G. candidum* was the same as when it was inoculated under the activated form, demonstrating a reduced specific production when it was inoculated under the freeze-dried form. As expected, PLA values increased and kept high until the end of the experimentation when *G. candidum* inoculum concentration under activated form was doubled. Experimentations conducted in both Ym synthetic medium and micro-malting assays showed that activating *G. candidum* before inoculation helps to launch its metabolism and permits the production of PLA during early stages of growth, leading to a higher reduction in T-2 toxin concentration. These findings support the hypothesis that the PLA is a primary metabolite produced during the early stages of growth [20].

The highest T-2 toxin reduction levels corresponded to the highest values of produced PLA, and this occurred between the second and third day of incubation time. In addition, the reduction in the T-2 toxin concentration in Ym synthetic medium makes it possible to achieve final concentrations lower than the European recommendation of 200 µg/kg. In the micro-malting assay, the T-2 toxin’s initial concentration is very high (12,000 µg/kg) when the fungal strain is inoculated alone for 5 days. Thus, the T-2 toxin reduction observed in experimentations where *G. candidum* was added does not agree with the European recommendation. However, these results are still very promising because the natural contamination levels of barley are much lower.

Based on the results obtained in the present study, the optimal culture conditions of *G. candidum* during the malting process were activating the yeast and increasing inoculum concentration to obtain better T-2 toxin concentration reduction. If the brewer wishes to maintain the inoculum at 0.2 g/L, the malting step should be reduced to a period of three days; otherwise, PLA values would decrease, leading to an increase in T-2 toxin levels. Nonetheless, if the brewer cannot afford to activate the yeast, the alternative is to double *G. candidum* inoculum concentration to guarantee the safety of the beer product. In this case, the malting step should also be limited to a duration of three days to avoid high T-2 toxin concentrations.

The results of this study demonstrate that there is a difference in the efficacy of *G. candidum* depending on the form in which it is used. The difference in efficacy between the freeze-dried and the activated form may be due to the freeze-drying process. Indeed, this latter can reduce microorganism viability, and finally, the activation step allows to overcome this reduction in viability by inoculating with a culture in an exponential growth phase. Moreover, storage conditions, such as temperature and duration of the freeze-dried yeast, are parameters that can influence cell viability. For instance, storage at temperatures ranging from ambient temperature to 40 °C was found to be harmful to dried yeast cells, which caused a 90% loss of viability per month. Thus, conservation of active dry yeast should be performed at a cool temperature and used as soon as possible [23].

Freeze-drying of biocontrol agents allows their stabilization and their conservation. Moreover, compared to liquid forms, dried products are easier to use in industrial processes. Specific procedures are required to guarantee long-term viability and metabolism stability. However, it may be considered as a source of stress for the microorganism itself [24]. For example, Abadias et al. (2001) tested the effect of freezing, freeze-drying process and the use of protectants on the viability of the biocontrol yeast *Candida sake.* They showed that liquid nitrogen freezing caused high deterioration levels to the cells with viability < 10% [25]. However, freezing cells at −20 °C could be a better alternative to the previous method as it registered around 30% of cell viability. When adding exogenous substances as protective agents such as lactose, glucose or fructose increased survival chances by 35% when added at 10% in an appropriate media [26]. A hypothesis suggests that liquid nitrogen quick-freezes cells immediately to a point where internal water freezes inside, causing membrane damage [27]. Therefore, it is advised to adopt rehydration and growth media enriched with these protective agents to increase yeast viability after freeze-drying [28]. In the case of *G. candidum,* used as a biocontrol agent to reduce T-2 toxin concentration, it is the capacity to produce PLA which is necessary. Thus, it is essential to ensure that its capacity to produce PLA is maintained at the same level after the freeze-drying process.

*G. candidum* was chosen among several species as a fermenting agent during the brewing process for many reasons: the production of thermostable hydrolytic enzymes (cellulase, β-glucanases, pentosanase) that facilitate the fermentation, flavoring of the malt due to the synthesis of amino acids, lowering of the pH and inactivation of the development of undesirable microflora. While it has many advantages, it is regrettable not to optimize its use to maximize the benefits and obtain a safe end product [29,30,31,32,33,34]. 

Furthermore, other factors can influence yeast growth and have an impact on the brewing process. Indeed, magnesium and zinc are key factors to improve the brewing process. They act as modulators of yeast stress as they maintain the stability and dynamics of the cell membrane. Studies on *Saccharomyces cerevisiae* showed that these metal ions are linked to the better viability of yeast during the brewing process [35]. They enhance signaling systems that allow a better response to environmental stress and decrease the permeability of the cell membrane [36]. Thus, zinc and magnesium-enriched rehydration medium might be one solution among others to increase the chances of active dry yeast viability.

## 4. Conclusions

In conclusion, important factors can influence the efficacy of *G. candidum* against *F. sporotrichioides* and *F. langsethiae* and T-2 toxin concentration, such as dehydration methodology, growth media and conditions, the addition of protective agents, rehydration conditions, stress induction and storage conditions to standardize and attain high levels of cell viability. All these elements should be taken into account during the brewing process to optimize the effectiveness of the biocontrol agent against these mycotoxigenic fungi to ensure a safe product. 

## 5. Materials and Methods

### 5.1. Reagents and Chemicals

T-2 toxin and phenyllactic acid (PLA) were purchased from Sigma-Aldrich (Saint-Quentin-Fallavier, France). Stock solutions were, respectively, prepared in dimethylsulfoxide (DMSO, Sigma-Aldrich, Saint-Quentin-Fallavier, France) and in acetonitrile–water (30:70 *v/v*) mixture and stored at −18 °C before use. Solvents used for T-2 toxin extraction and High-Performance Liquid Chromatography (HPLC) were of analytical grade quality and purchased from Thermo Fisher Scientific (Illkirch, France). Ultrapure water used for HPLC was purified at 0.22 µm by an ELGA purification system (ELGA LabWater, High Wycombe, UK).

### 5.2. Strains, Media and Culture Conditions

Two *Fusarium* strains were used: *F. sporotrichioides* 186 and *F. langsethiae* 2297. Both strains were isolated from contaminated barley kernels and were kindly provided by the French Institute of Brewing and Malting (IFBM). *Fusarium* spore solutions and pre-cultures were performed as described by Kawtharani et al. (2020) [20]. The filamentous yeast *G. candidum* X16010211 (IFBM Malting Yeast^®^) was purchased from DSM Food Specialties (La Ferté-sous-Jouarre, France) and supplied under freeze-dried form. In this study, *G. candidum* was used either under the freeze-dried form or under the activated form. For the latter, *G. candidum* was revivified before experimental use. For that, a 24 g/L of freeze-dried yeast was inoculated in 250 mL of yeast and malt (YM) liquid medium (glucose 5 g/L; yeast extract 1.5 g/L; malt extract 1.5 g/L; peptone 2.5 g/L pH 7) and incubated in an orbital shaker (New Brunswick™ Excella^®^ E24R—Eppendorf, Montesson, France) set at 22 °C at 150 rpm for 24 h. At the end of the incubation time, this culture was used as a starter culture set at 0.2 or 0.4 g/L, depending on the experiment.

Ym liquid medium was used during co-culture experiments. In order to mimic the malting conditions, micro-malting assays were performed. Therefore, microbial cultures were performed in 2 L bottles containing 200 g hulless barley kernels (JMT, Labège, France) submerged overnight in 200 mL distilled water (1:1 grain:water ratio) and sterilized 20 min at 121 °C before inoculation.

### 5.3. Co-Culture of Fusarium Strains and G. candidum in Ym Synthetic Medium

For co-culture experiments, Erlenmeyer flasks containing 150 mL of Ym medium were inoculated with *G. candidum* either under the freeze-dried form or under activated form at the final concentration of 0.2 g/L or 0.4 g/L. Then, *F. langsethiae* 2297 or *F. sporotrichioides* 186 was inoculated at the final concentration of 10^6^ spores/mL in their respective flasks. Cultures were incubated in an orbital shaker set at 22 °C at 150 rpm. The inoculation rates were chosen on the basis of previous work [19,20], and the incubation temperature was set at 22 °C to bring it as close as possible to the environmental conditions of the malting stage in the brewing process. Several incubation times were tested: day 1, day 2, day 3, day 4, day 5 and day 7. At the end of the incubation, for all culture conditions, the total dry weight, PLA and T-2 toxin concentrations were measured. For control conditions, each microorganism was inoculated alone (pure culture) at the same concentrations. All experiments were conducted in duplicates.

### 5.4. Micro-Malting Assays

In order to simulate the first steps of the malting process (steeping and germination), two-liter bottles containing 200 g of barley kernels were submerged with 200 mL of distilled water and left overnight to allow grains to soak in water. The next day, bottles were sterilized at 121 °C for 20 min. After complete cooling, *G. candidum* was inoculated using either under the freeze-dried form or under activated form final concentration of 0.2 g/L or 0.4 g/L. Then, *F. langsethiae* 2297 or *F. sporotrichioides* 186 was inoculated at the final concentration of 10^6^ spores/g in their respective flasks. As for co-culture experiments, cultures were incubated at 22 °C. Two incubation times were tested: three days and five days. At the end of the incubation time for all culture conditions, PLA and T-2 toxin concentrations were measured. For control conditions, each microorganism was inoculated alone at the same concentrations. All experiments were conducted in duplicates.

### 5.5. G. candidum, F. sporotrichioides 186 and F. langsethiae 2297 Biomass Evaluation in Co-Cultures 

In order to evaluate microorganism growth during the incubation period in pure cultures and co-cultures, vacuum filtration was performed to determine the total dry weight (g/L) as previously described by Kawtharani et al. (2020) [20].

### 5.6. T-2 Toxin and PLA Quantification by HPLC-DAD

#### 5.6.1. T-2 Toxin Quantification 

For T-2 toxin quantification in microbial cultures in liquid Ym synthetic medium, at the end of the incubation period, cultures were filtrated through Nalgene™ Rapid-Flow™ Filters of 0.45 μm pore size (Thermo Fisher Scientific, Villebon-Sur-Yvette, France) to remove microorganisms. Then, filtrates were extracted with 70 mL of ethyl acetate and shaken overnight at 150 rpm. For T-2 toxin quantification in micro-malting experiments, 400 mL of ethyl acetate were used to extract the whole content bottles. Then, bottles were shaken on Universal Shaker SM 30 B Control Edmund Bühler^®^ (Thermo Fisher Scientific, Villebon-Sur-Yvette, France) set at 150 rpm overnight.

In both cases, the organic phase was recovered and evaporated until dryness under a rotavapor set at 60 °C. Samples were resuspended with 2 mL of acetonitrile/water (30/70, *v/v*) mixture and filtered through 0.45 µm PTFE syringe filters (Thermo Fisher Scientific). Samples were conserved at 4 °C until further analysis. T-2 toxin was analyzed by C18 Gemini column C18, 150 mm × 4.6 mm, 3 μm and a pre-column with the same characteristics (Phenomenex, Torrance, CA, USA). T-2 toxin was detected and quantified using an Ultimate 3000 HPLC system coupled with a diode-array detector (DAD) (Dionex/Thermo Fisher Scientific, Courtaboeuf, Les Ulis, France) according to the methodology previously described by Medina et al. (2010) [37]. Analysis was conducted at 30 °C and T-2 toxin was confirmed by its retention time (min) according to a standard (Sigma-Aldrich) and quantified by measuring the peak area according to a standard calibration curve with concentrations ranging between 0.2 and 500 μg/mL. The T-2 toxin deacetylated form, i.e., HT-2 toxin, was also monitored in all experiments, and its quantification was calculated according to a standard calibration curve with concentrations ranging between 0.2 and 500 μg/mL.

#### 5.6.2. PLA Quantification

For PLA quantification in microbial cultures in liquid Ym synthetic medium, 1 mL of culture media was withdrawn at each sampling time and filtrated through 0.45 µm PTFE syringe filters (Thermo Fisher Scientific) to eliminate microorganisms from the supernatant before injection to HPLC apparatus. Regarding micro-malting assays, PLA was quantified in the same extracts as for the T-2 toxin quantification. Analyses of PLA were performed using a Luna C18(2) column (5 µm, 250 × 4.6 mm) and a pre-column with the same characteristics (Phenomenex). The PLA was detected and quantified using HPLC-DAD according to the methodology previously described by Kawtharani et al. [20]. PLA quantification was calculated according to a standard calibration curve with concentrations ranging between 0.01 and 1 g/L. In micro-malting experiments, PLA concentrations are expressed in g/g.

### 5.7. Statistical Analysis 

First, the normal distribution of data was tested by the Shapiro-Wilk test. Then, one-Way ANOVA followed by Tukey’s multiple comparisons test was used to analyze the differences between control and co-culture conditions. Differences were considered to be statistically significant when the *p*-value was lower than 0.05. Graphical values are represented by mean ± standard deviation (SD). Two-Way ANOVA followed by Tukey’s multiple comparisons post hoc test was used to analyze all conditions compared to each other. Data with different letters are significantly different *p*-value < 0.05, and graphical values are represented by mean ± standard deviation (SD). The statistical analysis of data was carried out with GraphPad Prism 8 software (GraphPad Software, La Jolla, CA, USA). 

## Figures and Tables

**Table 1:** PLA specific production (g PLA/g dry weight) in Ym medium inoculated in different conditions (Two-way ANOVA, Tukey’s multiple comparisons post hoc test, data with different letters are significantly different *p*-value < 0.05). ND = not detected.

Incubation Time (Days)					
Inoculation Condition	1	2	3	5	7
0.2 g/L freeze-dried	ND	0.181 ± 0.038 ^a^	0.112 ± 0.004 ^a^	0.034 ± 0.001 ^a^	0.001 ± 0.0002 ^a^
0.4 g/L freeze-dried	0.175 ± 0.011	0.186 ± 0.004 ^a^	0.059 ± 0.003 ^b^	0.049 ± 0.008 ^a^	0.001 ± 0.0002 ^a^
0.4 g/L activated	0.136 ± 0.006	0.157 ± 0.01 ^a^	0.129 ± 0.002 ^a^	0.098 ± 0.008 ^b^	0.086 ± 0.001 ^b^

**Table 2:** PLA concentration (g/g) in micro-malting experiments (mean ± SD) (Two-way ANOVA, Tukey’s multiple comparisons post hoc test, data with different letters are significantly different *p*-value < 0.05). ND = not detected.

	*F. langsethiae* 2297		*F. sporotrichioides* 186	
	Incubation Time (Days)		Incubation Time (Days)	
*G. candidum* inoculation condition	3	5	3	5
0.2 g/L freeze-dried	0.29 (±0.00) ^a^	0.13 (±0.02) ^a^	0.30 (±0.01) ^a^	0.14 (±0.01) ^a^
0.4 g/L freeze-dried	0.49 (±0.03) ^b^	0.25 (±0.01) ^b^	0.43 (±0.01) ^b^	0.23 (±0.01) ^b^
0.2 g/L activated	0.49 (±0.01) ^b^	0.19 (±0.01) ^a,b^	0.50 (±0.01) ^b^	0.22 (±0.01) ^b^
0.4 g/L activated	0.73 (±0.02) ^c^	0.39 (±0.02) ^c^	0.72 (±0.02) ^c^	0.77 (±0.01) ^c^

## Data Availability

Not applicable.
